# Supplementary material for: Are carbon emissions trading and green financial instruments synergistic? -Comprehensive quantitative research based on content analysis
Source: PLoS One. 2024 Mar 7;19(3):e0298601. doi: 10.1371/journal.pone.0298601 (PMC10919601; doi:10.1371/journal.pone.0298601)
Supplement: S1 Data — (ZIP) [file pone.0298601.s001.zip › Data/地级市Z_F工作报告.zip!/░▓╗╒/╬▀║■╩╨/└┤╫╘╣1⁄2╓┌║┼í╛┬φ┐╦╩2╛▌═°í┐_2007.docx]

——2007年1月13日在芜湖市第十三届人民代表大会第七次会议上

市长 陈树隆

各位代表：现在，我代表市人民政府，向大会作政府工作报告，请予审议。请各位政协委员和其他列席人员提出意见。

一、“十一五”发展开局良好

2006年是“十一五”规划开局之年。全市上下以科学发展观统领发展全局，锐意进取，扎实工作，圆满完成了市十三届人大五次会议确定的主要目标任务，全市经济社会保持了又好又快的发展势头。

预计全市实现生产总值480亿元，按可比价格计算，比上年增长15.5%；财政收入82亿元，增长21.5%，其中地方财政收入33.4亿元，增长19.8%；完成全社会固定资产投资287亿元，增长30%；实现社会消费品零售总额139亿元，增长15.2%；居民消费价格上涨1.2%；实现进出口总额8.35亿美元，增长31.8%；城市居民人均可支配收入10840元，农村居民人均纯收入4394元，分别增长12.6%、10%；城镇新增就业5.3万人，城镇登记失业率控制在3.8%以内；人口出生率控制在10‰以内；单位生产总值能耗下降4 %；主要污染物排放总量减少2%。一年来的工作主要表现在：

（一）新农村建设扎实推进。全面实施都市型现代农业发展规划。成立了一批农业企业行业协会，组建了一批农村专业合作经济组织，培育了一批“执照农民”。市级农业产业化龙头企业新增25家。皖南现代农业综合服务中心开工建设。实施水稻产业提升、畜牧富民、渔业富民、农机富民等工程，发放粮食直补、良种补贴、农机购置补贴和林业重点工程补贴6093万元，农民生产积极性明显提高。战胜了秋旱、特大病虫害等多种自然灾害，全年粮食总产预计达80万吨，创历史新高。植树造林和绿色长廊建设均获全省第一。

坚持市县一体、城乡统筹发展。启动市域农村居民点及新型社区布点规划编制工作，实施“百村整治、十村示范”工程，推进公共设施和公共服务向农村延伸覆盖。芜南路拓宽改造和南陵供水项目正式启动。市县快速通道芜屯路建成通车。全市“村村通”工程建成水泥路650公里，繁昌县在全省率先实现“村村通”目标。新开通城乡客运公交化线路63条。万亩以上圩口堤防达标工程完成年度任务。农村安全饮水工程进展加快，当年解决了2.3万农民吃水难问题。实施了三县天然气工程。农村改水改厕工作稳步推进。南陵县、繁昌县实施新型农村合作医疗试点，受惠农民70万人。加快农村保障体系建设，2.4万被征地农民基本生活得到保障。全面推进农村综合改革，农村基层工作新机制初步建立。市级多渠道筹资8.8亿元，支持“三农”发展。

（二）工业经济加速发展。全市完成工业投资123.6亿元，同比增长35.9%，占全部固定资产投资的43%。奇瑞三期、海螺水泥熟料基地、新兴铸管扩能、华电芜湖电厂一期、响水涧抽水蓄能电站等一批重大项目顺利实施。预计全市实现工业增加值240亿元，比上年增长22.1%。汽车及零部件产业快速增长，实现产值206亿元，同比增长73.2%。奇瑞汽车年产量达30.5万辆，名列全国乘用车销量第4。海螺、楚江、鑫科等一批骨干企业快速发展。全市规模以上工业企业达750家，比上年末增加120家；年销售收入超亿元企业达74家，比上年末增加10家。全市规模以上工业企业净利润增长50%，经济效益综合指数为205%，比上年提高12.2个百分点。

以科技创新引领工业发展。争取国家级科技计划项目28项，省级项目36项，国家和省科技支持资金1.2亿元。芜湖经济技术开发区被授予国家汽车及零部件出口基地、国家级汽车电子信息产业园。长信科技、鑫龙电器被批准为国家级高新技术企业，27家企业被认定为省级高新技术企业。“奇瑞”被认定为中国驰名商标。华东光电所被批准为全国专利试点企业。全市专利申请量超过800件，同比增长100%。去年市本级筹资14亿元，支持工业发展和园区基础设施建设。

（三）第三产业保持良好发展态势。物流业发展加快。裕溪口煤码头基本建成，汽车滚装码头、朱家桥集装箱码头开工建设。全市港口货物吞吐量达3800万吨，集装箱运量达10万标箱，比上年增长62%。城乡商业基础设施建设力度加大。侨鸿国际购物中心、汉爵阳明大酒店等项目建成开业，美食街二期、银湖波尔卡、欧尚超市等项目顺利实施。“万村千乡”市场工程稳步推进，新建配送中心3个、农家店377个。我市被吸纳为长三角旅游合作组织成员城市。争创了王稼祥纪念园等3个4A级旅游景区、陶辛水韵等3个全国工农业旅游示范点。华强旅游城建设进展顺利。建成了会展中心，成功举办了科博会、汽博会、房博会、青博会等专业会展。

（四）改革开放取得新突破。较好地解决了企业改制的遗留问题。围绕企业上市，新设了6家股份有限公司。深化社区管理体制改革，推行社区“议行分设”，市区街道办事处由34个整合为27个，社区由146个整合为94个。

对外开放进一步扩大。继续把招商引资作为经济工作的重中之重，开展有针对性的招商活动，引资工作取得了重大进展。全市合同利用外资3亿美元，比上年增长118%；实际利用外资3.4亿美元，增长32.3%；实际利用内资215亿元，增长44.5%。引进内资1亿元以上的项目40个，外资1000万美元以上的项目15个，世界500强企业5家。经济技术开发区招商取得突破，台达电子、沃特新材料、江森自控、德尔福等一批大项目进区发展。县区招商成效明显，宝成鞋业、全息浮显、巨龙液压等一批大项目成功落户。对外贸易快速增长，全年出口6.24亿美元，比上年增长35%，其中机电类产品占出口总额70%。企业“走出去”步伐加快，全年新批企业境外投资项目5个。

（五）财政金融支持发展力度加大。重新配置市、区财权和事权，有效衔接省管县财政体制，实现了新老财政体制的平稳过渡。把握国家财税体制改革和支持中部崛起的政策机遇，合理组织财政税收，优化了收入结构。获得国家和省多种财政专项资金8.7亿元。市建投公司获国家开发银行贷款授信40亿元。中部地区首家农村商业银行芜湖扬子农村商业银行挂牌运营。全市金融机构人民币各项存款余额462.4 亿元，比年初增加80亿元；各项贷款余额386.3亿元，比年初增加42.2亿元。域外金融机构对我市贷款余额72亿元，比年初增加8.7亿元。全市小企业贷款余额83.6亿元，比年初增加12.5亿元。

（六）城乡规划建设管理工作取得新进展。完成行政区划重大调整，编制了城市总体规划纲要和相关专项规划。建设了一批重大交通基础设施项目，沿江高速繁昌段主体工程基本完成，铜南宣高速、芜雁高速、芜申运河工程开始启动。滨江公园和临江桥建设进展顺利。银湖路刚改柔试验及绿化改造工程完成。三环路、经六路、东一路、东四大道等道路建设步伐加快。整治背街背巷177条。改造了部分慢车道、人行道。加速推进市区天然气二期工程，新增居民用户1.2万户。调整优化城区公交线路，公交服务得到改善。保兴垾水系综合整治工程开工建设。朱家桥污水处理厂建成投入使用。市区水域补水、城南水系整治工程完成年度任务。新增城市绿地24万平方米。集中整治“四乱”行为，清理整顿户外广告，市容管理取得明显成效。去年市级多方筹资28亿元，加快城乡基础设施建设。

（七）人民生活进一步改善。加大了就业工作力度。出台了支持全民创业的实施意见，降低了创业门槛，推动一批特色创业基地建设，发放小额贴息贷款7136万元。出台了托底就业政策，安置就业困难人员1.1万人。投入“以奖代补”资金5600万元，免费培训1.62万人次，安置下岗失业人员2.1万人。实施农村劳动力转移培训“阳光工程”，当年培训1.6万人。全市新增个体工商户近6000户，私营企业1800多户。进一步提高社会保障水平。开展了全市“签约参保”大检查，养老、医疗、工伤和生育保险参保人数分别比上年增加3.6万人、1万人、3.7万人和2.3万人，是新增人数最多的一年。提高了最低工资、养老金和失业金标准，增幅分别达35%、33%和23%，是历年来调整幅度最高的一次。去年全市养老保险基金征缴收入7亿元，同比增长32%。医疗保险降低个人负担比例2个百分点。进一步完善城乡社会救助体系。加强对因病致贫等特困群体的生活救助。提高城乡居民最低生活保障标准，保障了全市7.3万城乡低保对象的基本生活。出台了房地产调控政策具体实施意见。加大力度清收各类土地出让金。加快拆迁安置房、廉租房建设，全年竣工拆迁安置房25万平方米，其中廉租房1.3万平方米。商品房销售面积148万平方米，增长21.7%。市区住宅商品房销售均价每平方米3295元，上涨4.1%，低于全国平均涨幅。完善住房公积金制度，公积金归集、贷款和还贷购房提取额分别达6.9亿元、7.1亿元和3.8亿元。

（八）各项社会事业全面进步。各级各类教育进一步发展。芜湖职业技术学院进入首批国家示范高职院校建设计划。芜湖教育学院改制为芜湖信息技术职业学院。芜湖技师学院经省政府批准成立。繁昌县代表安徽省顺利通过“两基”巩固提高国家验收。促进义务教育均衡发展，公办学校中小学生一律实行免试就近划片入学。加强薄弱学校建设，实施均衡发展项目81个。建立资助农村学生“职业教育券”制度，维护了教育公平。完善城乡公共卫生和医疗服务体系。市疾病预防控制中心、红十字紧急救援中心和传染病医院建成投入使用。开展了医院管理年活动，打击非法行医，查处医药销售领域的商业贿赂行为。大力发展文化事业和文化产业。组建了芜湖日报报业集团、市广电总台和市艺术剧院。繁昌人字洞被确定为全国重点文物保护单位。芜湖铁画首批进入国家非物质文化遗产名录。以社区文化艺术节、“杜鹃花工程”、送文化下乡活动为载体，群众性文化活动蓬勃开展。点校出版《芜关榷志》，编纂了芜湖文化丛书，《芜湖年鉴》获全国一等奖。推动群众体育和竞技体育协调发展，省运会我市获良好成绩，世界杯跳水赛我市运动员获1枚金牌。开展了第二次全国农业普查工作。市档案馆通过国家综合档案馆省一级认定。去年市级用于科、教、文、卫等社会事业发展资金达11亿元，同口径比上年增长58.2%。

努力促进人口、资源、环境与经济协调发展。加大国家环保模范城市创建力度，推进“百镇千村万户”生态示范工程建设。开展城镇建设用地增加与农村建设用地减少相挂钩试点工作，加强土地开发整理，保障了重点项目用地需要。完善经营性用地管理，强化责任考核，有效保护了耕地。全面整顿和规范矿产资源开发秩序，地质灾害防治工作取得成效。扎实开展出生人口性别比综合整治，我市被评为全国农村部分计划生育家庭奖励扶助制度工作先进市。

进一步加强社会管理。落实安全生产责任制，推行风险抵押金制度，开展重大危险源普查登记和分类分级管理，加强安全隐患整治，关闭了一批不具备安全生产条件的高危企业。加强信访工作。扎实推进社会治安综合治理，严厉打击严重刑事犯罪和经济犯罪，全市发案率比上年下降5.4%，破案率上升28.5%。“双拥”工作和国防后备力量建设进一步加强。妇女儿童、老龄、残疾人事业进一步发展。民族、宗教、气象、外事、侨务、对台工作取得新成绩。

（九）政府自身建设得到加强。全面推进依法行政，认真执行市人大及其常委会决议，重大问题广泛征求人大代表、政协委员意见，自觉接受法律监督和民主监督，全年办理人大代表议案、建议93件，政协委员提案419件。出台了优化发展环境和加强效能建设的决定。全面清理审批项目和行政事业性收费项目。优化审批流程，市行政服务中心全年办理各类审批事项57万余件。加强政府招标采购管理，完成政府性投资招标1250项。强化政务督办。推进政务公开，完善政府新闻发布制度。建成电子政务安全中心、数据交换中心一期工程，初步建立了覆盖全市的电子政务专网。加强政民互动，改版了市政府门户网站，“市民心声”入选中国地方政府创新奖。认真落实党风廉政建设责任制，加大反腐倡廉工作力度，加强行政监察和审计监督，严肃查处违纪违法案件，从源头上防治腐败。

各位代表，以上成绩是在宏观发展环境偏紧、市场约束因素较多、行政区划调整较大的情况下取得的，确实来之不易。这是市委坚强领导的结果，是市人大及其常委会和市政协监督支持的结果，是全市人民团结奋斗的结果。在此，我谨代表市人民政府，向全体人大代表、政协委员，向全市广大工人、农民、知识分子、干部、驻芜解放军指战员、武警官兵和政法干警，向各民主党派、人民团体，向关心、支持芜湖发展的各界人士，表示衷心的感谢和崇高的敬意！

我们也清醒地看到，工作中还存在着困难和问题，主要是：经济结构还不够优化，支柱产业和龙头企业竞争力还不够强，又好又快发展的基础还不够稳固；招商引资工作进展还不平衡，民营经济发展还不够快，全民创业的社会氛围还不够浓；社会事业发展还显不足，社会保障水平还不高；城市功能还不够完善，建设力度还要进一步加大；政府服务与人民群众的要求还有距离，发展环境有待进一步改善。我们将高度重视这些问题，采取切实有效措施，努力加以解决。

二、2007年工作总体要求和工作目标

今年是深入贯彻落实科学发展观，推进和谐芜湖建设的重要一年，做好今年工作，具有十分重要的意义。当前我们正处于可以大有作为的重要战略机遇期，正处于实现跨越式发展、争当排头兵的关键时期。我们既面临着国家实施中部崛起战略、支持皖江城市带发展、支持自主创新、推进社会主义新农村建设、外资及东部产业加速向中部转移等难得的历史性机遇，也面临着宏观经济环境不确定因素增多、产业加速分化重组、区域竞争激烈、社会矛盾凸显等诸多挑战。我们要进一步增强使命感和紧迫感，坚定信心，开拓创新，奋发进取，切实做好各项工作。

今年政府工作的总体要求是：认真贯彻党的十六届五中、六中全会，中央经济工作会议和省、市第八次党代会精神，全面落实科学发展观，突出更好更快发展主题，坚定不移地实施工业强市、自主创新、统筹发展、东向发展战略，着力调整经济结构和转变增长方式，着力加强资源节约和环境保护，着力推进改革开放和自主创新，着力促进社会发展和解决民生问题，加速工业化、城市化、“三农”现代化进程，不断提高人民生活水平，努力构建和谐芜湖，为在全省率先全面实现小康打下坚实基础。

经济社会发展的主要预期目标：全市生产总值增长14%以上，财政收入与经济同步增长，全社会固定资产投资增长20%以上，城市居民人均可支配收入增长10%，农村居民人均纯收入增长6%。主要控制指标：城镇新增就业5万人以上，城镇登记失业率控制在4%以内，人口出生率控制在12‰以内，单位生产总值能耗下降4%，主要污染物排放总量减少2 %。

实现上述目标，任务十分艰巨。我们必须进一步增强工作的前瞻性、创造性、系统性、坚韧性和紧迫性，立足科学发展、和谐发展、率先发展，充分发挥我市承东启西的区位优势和交通便捷的枢纽优势。继续大力推进产业结构调整和优化升级，做大做强支柱产业，形成产业发展优势；继续大力推进空间布局规划调整和重大基础设施项目建设，提高组团式发展水平，形成城乡一体化发展优势；继续大力推进各项改革，创新体制机制，形成制度优势；继续大力推进和谐芜湖建设，加快实施民生工程，进一步完善激发活力、平衡利益的制度体系，形成团结和睦、凝心聚力推进跨越式发展的社会环境优势；继续大力推进创新文化、和谐文化、廉政文化建设，构建具有芜湖特色的价值观，形成先进文化优势，抢占全省乃至整个中部地区竞争的制高点，长期保持经济社会又好又快的发展势头，实现经济社会发展的新跨越。

三、全力推进经济更好更快发展

把加快发展作为第一要务，紧紧扭住经济建设这个中心不放松，以结构调整为主线，以项目建设为抓手，以改革开放为动力，依靠科技进步，转变经济增长方式，夯实加快发展的物质基础。

（一）大力实施工业强市战略，努力打造先进制造业基地

坚持有限目标，有所为，有所不为，引导企业通过观念、机制、技术和品牌的创新，迅速做大做强，实现高质量、高效益、高速度和集群化发展。促进大企业大集团快速发展，继续壮大支柱产业。大力发展汽车产业，努力把奇瑞公司建设成为具有国际竞争力的优势企业。积极创造条件，推动海螺集团通过资本市场筹集资金，加大在芜项目投入。推进新兴铸管无缝钢管、沃特新材料等重点项目建设，提升材料产业整体水平。推进台达电子等项目建设，带动形成电子产业集群。推进华电芜湖电厂一期、响水涧抽水蓄能电站项目建设，做好新电源点项目前期工作。通过加速企业改制、吸引增量嫁接、运用高新技术和先进适用技术改造等多种手段，加快传统产业升级步伐。实施好“百千工程”，重点扶持成长性好，拥有“专、精、特、新”产品的中小企业，促进大中小企业协调发展。以示范企业和项目为重点，推进制造业信息化工程重点城市建设，以信息化带动工业化，以工业化促进信息化。引导企业走品牌发展之路，争创国家、省级名牌产品。

（二）大力实施统筹发展战略，扎实推进新农村建设

按照城乡产业发展、规划管理、基础设施、社会事业、就业和社会保障、生态环境建设一体化的要求，加快基础设施和公共服务向农村延伸覆盖，推进新农村建设。一是用工业理念发展现代农业。加快实施都市型现代农业发展规划，培育壮大优质稻米、生态良种畜禽、名特优新水产、高档苗木花卉、绿色蔬菜与食用菌、特种经果林与中药材等主导产业。通过以奖代补等多种方式，扶持农业产业化龙头企业加快发展。推广“酵母工程”，培育“执照农民”，发展适度规模经营。组织实施好农业科技入户、综合信息服务、测土配方施肥等项目。加快皖南现代农业综合服务中心建设。二是完善农村基层工作新机制。深化农村综合改革，完善为民服务全程代理制和公共服务平台运行机制，引导、支持农民专业合作经济组织加快发展。实施农村劳动力“阳光工程”培训和技能培训。三是努力改善农村生产生活条件。完成市域农村居民点建设布点规划，继续实施村庄整治工程。推进芜南路拓宽改造和南陵供水工程。完成农村公路“村村通”工程。加快县城防洪、万亩以上圩口堤防达标、大型泵站改造项目建设进度，实施一批农田水利、水土保持、农业综合开发项目。四是加速县区经济发展。加快各具特色的县区经济开发区和镇级产业集中区建设步伐。加大市级财政支持县区经济发展的力度。进一步完善市、区两级财政体制，增强区级经济发展动力。加大协调力度，增强县区融资能力。强化市直部门责任，支持县区发展。

（三）发挥商埠城市优势，加快发展现代服务业

大力发展物流业。支持芜湖港储等重点物流企业推进基础设施建设，打造沿江多式联运枢纽。强化政府规划引导，重点发展集聚力强、规模大、档次高的专业性市场。继续实施“万村千乡”市场工程。建设美食街二期、小九华商业街等特色街区。建立发展引导资金，推动现代服务业加快发展。改善金融生态环境，加快金融业发展步伐。积极引进域外金融机构来芜设立分支机构。推行外部信用评级制度，加快建立中小企业征信体系，建设“信用芜湖”。通过对接座谈、银团贷款、完善担保等方式，促进银政、银企合作。建设公用事业收费信息平台，解决群众缴费难问题。推进企业改制上市进程，做好精诚铜业挂牌上市工作，支持海螺、鑫科等企业定向增发融资，争取市建投公司发行企业债券。培育担保、典当、风险投资业，发展会计、律师、咨询等中介服务业和各类行业协会。大力发展旅游经济，整合市域旅游资源，加强景区建设，提升接待服务能力和管理水平。推进华强旅游城项目建设，力争年内建成开园。办好特色品牌会展，做大做强会展经济。

（四）高起点做好城乡规划建设管理工作，完善城市功能

适应区划调整要求，完善城市总体规划，做好市域中心城区、副中心城区、中心镇区规划调整工作，整合资源比较优势，规划建设各具特色的功能分区。编制重要地块控制性详规和相关专业专项规划。

大力推进城市基础设施建设。加快城市东扩建设步伐，加速东区道路设施建设，开工建设商务文化中心，实施神山公园环境整治。加快城区主干路网建设步伐，加速滨江公园工程建设，建成三环路中段、扁担河大桥，拓宽改造弋江路，推进赤铸山东路、东四大道延伸段、经六路、纬三路、纬四路、沿河路、神山东路、神山南路等主要道路建设，改造一批市区慢车道、人行道。加快铜南宣高速、芜雁高速、芜申运河等重点工程建设。

继续推进相对集中行政处罚权工作，强化城市管理。发挥街道、居委会基础作用，抓好“门前三包”，加强建筑垃圾与工程渣土管理，巩固“四乱”整治成果，形成长效管理机制。实施市区户外广告专项整治。推进城市管理信息系统建设。

（五）深化体制改革，提升外向型经济发展水平

稳步推进各项改革。做好重点企业改制重组工作。落实推动全民创业的各项政策措施，优化发展环境，放手发展民营经济。制定建设规划，加快新材料、光电子等特色创业基地建设。完善政府部门、金融机构、中介服务机构联席会议制度，强化全民创业服务。深化以审批制度为重点的行政管理体制改革，全面推进“两个集中”，提高审批效率。清理经营服务性收费，减少收费项目，降低收费标准。以深化人事制度和产权制度改革为重点，加快事业单位改革步伐。积极推进教育、卫生管理体制改革，推动社会资本投资教育、卫生领域。

提高招商引资工作水平。围绕汽车及零部件、材料、电子电器产业，着力引进国内外大企业大集团来芜发展。继续推进小分队招商，强化责任管理，深入重点地区，开展驻点招商。积极推进以外引外，以商招商。加强招商载体建设，完善各类开发区、工业园区设施功能，提高承载能力，促进产业集聚。发展总部经济，吸引国内外大企业大集团来芜设立生产基地、研发中心、营销总部和地区总部。转变外贸增长方式，优化出口产品结构，大力引进国外先进技术设备。实施“走出去”战略，支持企业拓展国际市场，开展跨国经营。

（六）大力实施自主创新战略，加强创新能力建设

建设创新型城市。弘扬“崇尚创新、宽容失败、支持冒险、鼓励冒尖”的创新文化，营造创新创业的社会氛围。切实重视企业研发能力建设，加大政策支持力度，积极引导企业加大研发投入。支持创新型企业建立重点实验室、工程技术研究中心、企业技术中心等研发机构，加快国家节能环保汽车工程技术研究中心、国家特种显示工程技术研究中心项目建设。落实国家知识产权试点城市工作方案，争创一批全国知识产权示范创建企业。完善高新技术创业服务中心功能，加快汽车电子及关键零部件企业孵化器项目建设。

努力构建“人才高地”。加大人才培养工作力度，积极为创新人才施展聪明才智创造良机、搭建舞台，造就一批高层次创新人才团队。加强人才引进工作，创造条件，重点引进各类高层次、创业型人才。建设好留学人员创业园、软件园、火炬创新创业园、科普产业园等创新创业平台。维护企业家合法权益，培养更多具有创新精神的企业家。

四、努力促进社会更加和谐

把加快实施民生工程、促进社会和谐作为政府工作的重中之重，着力解决人民群众生产生活中的困难和问题，努力促进社会公平正义，构建和谐芜湖。

(一) 千方百计扩大就业

实施“创业扶持工程”，建设一批高标准的创业园、街，加大小额贷款发放力度，鼓励创业，以创业带动就业。抓住全国城乡统筹就业试点机遇，积极争取政策和资金支持，推进“蓝领工程”建设。整合资源，加快芜湖技师学院建设步伐，落实好“首席技师”制度，提高就业服务水平和就业市场化程度。加大劳动关系协调力度，依法完善用工、裁员、民工薪酬制度，强化劳动保障执法监察，维护用人单位和劳动者双方的合法权益。

（二）不断提高人民生活水平

认真落实国家粮食直补、良种补贴、农机购置补贴等各项支农惠农政策，增加农民收入。规范“一事一议”筹资筹劳制度，确保农民负担不反弹。落实相关政策，改善失地农民生活。实施公务员工资制度和机关事业单位津补贴改革。调整住房供应结构，多渠道筹措建设资金，加大拆迁安置房、廉租房建设力度，着力解决低收入家庭住房困难。加强房地产开发用地管理，健全房地产统计和信息披露制度，开展房地产交易秩序专项整治。完善住房公积金制度，维护职工权益。

（三）进一步完善社会保障体系

高度重视社会保险“扩面提率”工作，继续完善养老、失业、医疗、工伤、生育保险政策，让更多的社会成员享受社会保障，提高社会保障水平。制定事业单位养老保障办法，为事业单位改革创造条件。开展农村养老保险试点。完善社会保险一票征缴机制，多渠道筹集社会保险基金，做好提高社会保险统筹层次工作，确保社会保险资金按时足额社会化发放。积极发展社会救助和社会慈善事业，加强城市流浪乞讨人员救助工作，动员社会力量扶贫帮困。关心、支持老龄和残疾人事业。做好未成年人保护工作，开工建设儿童福利院。

（四）大力发展教育、卫生、文化事业

以提升市民素质、维护教育公平为目标，大力发展教育事业。认真落实义务教育法，推进公共教育均衡发展，加强教师队伍建设，深化素质教育。挖掘优质高中资源，加快市职教基地和三县职教中心建设，积极发展职业教育，提高高中阶段入学率。加快高教园区配套设施建设，支持在芜高校发展。完善中小学布局规划，重点加强农村中小学规划调整工作。加大薄弱学校建设投入，启动中小学办学水平标准化建设工程。市十二中建成投入使用。保障进城务工人员子女享受公平教育权利。

整合医疗卫生资源，加强城乡医疗卫生服务体系建设，逐步建立覆盖全体居民的卫生服务网络。做好市属三县创建省农村卫生示范县工作。加快弋矶山医院病房大楼、市二院门诊楼建设进度。完善医疗急救网络，提高疾病预防控制和突发公共卫生事件应急处置能力。

以打造文化强市为目标，大力发展文化事业和文化产业。制定文化发展总体规划和促进文化产业发展的意见。深化文化体制改革，推动艺术剧院、大众影都等文化载体的市场化运作，加快中国铁画馆、徽派建筑艺术博物馆、银波艺术产业园等项目建设。市图书馆、群艺馆、博物馆建成投入使用。开工建设文化艺术中心。积极开展群众文化活动，加快文化事业发展步伐。大力推进农村广播电视“村村通”工程建设。组织开展全民健身活动，努力提高竞技体育水平。办好全省第五届农民运动会。加强档案信息化建设和开发利用。搞好市志、芜湖通史和文化丛书编修。

（五）建设资源节约型、环境友好型社会

发展循环经济，加快新兴铸管等企业资源综合利用项目建设。推进工业节能降耗，实施恒鑫等企业节能技改工程。制定节能降耗实施意见，进一步落实资源节约工作责任制。

保护和合理开发利用资源。加强土地调控，清理存量划拨用地，保障重点项目用地需要。提高投资强度，节约使用建设用地。完善责任机制，加强耕地保护，加大土地开发、复垦整理力度，确保耕地占补平衡。做好土地利用总体规划修编工作。开展城镇建设用地增加与农村建设用地减少相挂钩试点。完善国有资源有偿使用制度，加强矿产资源整合，依法保护、合理开发自然资源。

推动生态市建设。推进南陵县、芜湖县、弋江区3个国家生态示范区试点工作。实施农村小康环保行动计划和“百镇千村万户”生态示范工程。加强林地、湿地保护。推动绿色长廊二期工程等林业重点工程建设。推进集体林权制度改革，加强森林资源管护。加速国家环境保护模范城市创建步伐。加快医疗垃圾集中处置和城市生活污水处理等设施建设，扩建朱家桥污水处理厂，启动城南污水处理厂一期工程。加大环保执法工作力度，继续开展环保整治专项行动，加强对重点污染企业的监管。

稳定低生育水平，提高出生人口素质。强化流动人口计生服务管理，有效治理出生人口性别比问题，建立依法综合治理长效工作机制。

（六）努力营造民主法治的社会环境

建设和谐文化。以社会主义核心价值体系引领社会思潮，加强思想道德建设，注重人文关怀，促进社会成员心理和谐，塑造自尊自信、理性平和、积极向上、开放包容的社会心态。开展民主管理示范创建活动，完善基层民主管理。进一步畅通民主渠道，切实做好信访工作。创新社区管理体制，推进社区自治，夯实社会稳定和谐的微观基础。支持各类组织、企业和个人开展社区服务，探索农村居民点及新型社区建设的新途径。深入开展“五五”普法，做好法律服务和法律援助工作。加强人民防空和国防后备力量，支持驻芜部队建设，深入开展国防教育和双拥共建活动，争创“全国双拥模范城”。加强民族、宗教和侨务工作。保障妇女儿童权益。

构建“平安芜湖”。加快社会治安防控体系建设，及时排查化解矛盾纠纷，坚决打击各类犯罪活动，维护社会稳定。整合应急资源，加强应急平台建设，健全处置各类突发性事件的管理体制和工作机制。加强矿山、交通等重点行业安全监管，开展安全隐患专项整治，防止重大事故发生。

（七）实施好民生工程

促进社会和谐，关键是要解决好人民群众最关心、最直接、最现实的利益问题。今年将集中财力，实施好以下23项民生工程：完善城乡居民最低生活保障制度；提高农村“五保户”生活水平；建立健全城镇养老保障体系；完善农村部分计划生育家庭奖励扶助制度；全面实施新型农村合作医疗制度；探索建立覆盖城镇居民的医疗保障制度；提高民政救助对象大病医疗救助水平；强力推行高危行业职工工伤保险制度；建立健全重大传染病病人医疗救助和生活救助保障机制；启动惠民医疗工程；加强农村卫生和城市社区卫生服务体系建设；建立完善城乡就业服务体系；强力推进“签约参保”工作；全面实施城乡义务教育经费保障机制改革；用两年时间全面消除城乡中小学D级危房；加速推进农村饮水安全工程；深入开展市区环境综合整治工程；推进保兴垾综合整治工程；加快廉租房建设；启动芜湖古城改造工程；大力实施食品药品安全放心工程；进一步推进“平安芜湖”建设；实施文化惠民工程。

五、着力建设人民满意政府

坚持执政为民，切实转变政府职能，不断提高依法行政、民主行政、科学行政的能力，努力建设法治政府、服务政府、效率政府、廉洁政府。

提高政府依法行政水平。自觉接受人大及其常委会监督，认真落实人大及其常委会的决定，及时向人大及其常委会报告工作。积极支持人民政协履行政治协商、民主监督、参政议政的职能，主动听取各民主党派、工商联、无党派人士及各人民团体的意见和建议。认真办理人大议案、代表建议和政协委员提案。贯彻国务院《全面推进依法行政实施纲要》。建立健全公众参与、专家论证和政府决定相结合的行政决策机制，推进决策科学化、民主化。完善社会公示、听证和政府新闻发布等制度，推进政务公开。重视新闻舆论监督，加强“市民心声”和政府网站建设，自觉接受社会监督。

加快转变政府职能。运用经济手段和法律手段加强经济管理。强化市场监管职能，为市场主体创造公正、公平的发展环境。加强社会管理职能，健全公共服务体系。完善公共财政体制框架，调整支出结构，把更多的财力投向公共服务领域。加强公务员学习和依法办事能力建设，努力造就一支高素质的公务员队伍，提高行政管理水平。

加强行政效能建设。全面推行行政执法责任制、评议考核制、限时办结制、行政问责制。优化工作流程，提高行政效率。大力推进电子政务建设，加强政民互动。着力增强政府的公信力和执行力，严肃整治行政不作为和乱作为，切实解决人民群众反映的突出问题。加强政务督查，确保政令畅通。加强制度建设，逐步形成与政府管理创新相适应的激励约束机制和社会化评价机制，以制度管人管事。

坚持从严治政，廉洁从政。认真贯彻落实《建立健全教育、制度、监督并重的惩治和预防腐败体系实施纲要》，深入推进反腐倡廉工作。完善政风评议制度，坚决纠正损害群众利益的行为。开展行政系统“理想、责任、能力、形象”教育，厉行勤俭节约，力戒铺张浪费，反对奢靡之风。进一步强化对权力运行的制约和监督，认真落实用人、用钱、用地、用权的各项制度，加强行政监察和审计监督，强化领导干部任期经济责任审计，建立健全从源头上防治腐败的体制机制。

各位代表，芜湖要实现更好更快的发展，需要我们付出更大更艰苦的努力。让我们在中共芜湖市委的坚强领导下，全面贯彻落实科学发展观，紧紧团结和依靠全市人民，解放思想，凝心聚力，顽强拼搏，开拓进取，努力开创科学发展、和谐发展、率先发展的新局面，以优异的成绩迎接党的十七大胜利召开！
